# An Incidental Case of Morgagni Hernia With Beaver Tail Liver in an Adult

**DOI:** 10.7759/cureus.49769

**Published:** 2023-12-01

**Authors:** Ruthwik B Shankar, Adheep B Amberker, Vidyasagar Belgundi

**Affiliations:** 1 Pulmonary Medicine, JJM (Jagadguru Jayadeva Murugarajendra) Medical College, Davanagere, IND

**Keywords:** eventration of diaphragm, congenital diaphragm hernia, lung, beaver tail, diaphragm, morgagni hernia

## Abstract

Morgagni hernia is a rare congenital defect of the diaphragm, especially seen in children but rarely observed in adults. It occurs due to a congenital defect during the development of the diaphragm. Bochdalek hernia is a common congenital form of diaphragmatic hernia. Morgagni hernia is usually rare with a prevalence of approximately 2-3%. Beaver tail liver, which is also called sliver of liver, is a rare variant of liver morphology. Sometimes elongated left lobe of the liver can extend laterally across the midline to contact and often surround the spleen.

A 46-year-old female from Karnataka complained of fever with chills and lower back aches for seven days with no history of chest pain, vomiting, or diarrhea. There have been no similar complaints in the past. She had no other comorbidities. She was a non-smoker and non-alcoholic. Biomass gas exposure for 20 years was noted as she cooked food with firewood. She was a housewife by occupation with no history of trauma or surgeries in the past. The general physical exam was unremarkable. The respiratory system was normal. Auscultation showed decreased breath sounds in the mammary area of the right side of the chest with normal vesicular breath sounds in all other areas. Per abdominal exam showed a flat abdomen. Umbilicus was central in position. There was tenderness in the right hypochondriac and epigastric regions with no guarding or rigidity. On examining the cardiovascular system, apical impulses could not be palpated and normal heart sounds were heard with no cardiac murmurs. Other systems examination was normal. Routine blood investigations were done, revealing hemoglobin of 11.6%, total WBC of 6270 cells, and hematocrit of 33.1%. The renal function test was within normal limits (creatinine = 0.7 mg/dl).

A chest X-ray revealed a right lower zone, para cardiac well-circumscribed structure suggestive of a cyst with an air-fluid level inside. Chest CT was suggestive of a hernia in the anterior aspect of the diaphragm measuring 3.5 x 3.3 cm at the level of D9 vertebral body with transverse colon and omentum as its contents, ascending upwards into anterior and superior mediastinum for a length of 13 cm causing shift of cardia posteriorly and to the left (anterior midline diaphragmatic hernia - Morgagni hernia). A hyperdense lesion (Hounsfield unit = 64) measuring 1.3 x 1.8 cm was noted in segment seven of the right lobe of the liver, suggestive of a complex cyst. Beaver tail was noted in the liver.

Morgagni hernia usually presents in younger age groups with respiratory symptoms. Its incidental detection in adults is very rare. In this case, the patient was having lower backache and no other gastrointestinal symptoms. The respiratory and cardiothoracic systems get affected because the intestinal contents herniating through the diaphragm shift the position of the cardia and the lower lobes of the lungs, which may have implications such as repeated cough and infections. Symptomatic hernias are usually detected in an early age group. It can present with symptoms of gastrointestinal obstruction or acute chest symptoms or can even be asymptomatic. Treatment is primarily surgical repair of the hernia. This can be done either transthoracically or transabdominally. It is usually advised that surgical repair should be done even in asymptomatic cases as in this case, to avoid obstruction of the intestine or worsening of the hernia that is pulling the abdominal contents into the thorax.

## Introduction

Morgagni hernia is a rare congenital defect of the diaphragm, especially seen in children but rarely observed in adults. It occurs due to a congenital defect during the development of the diaphragm [1]. Bochdalek hernia is a common congenital form of diaphragmatic hernia. Morgagni hernia is usually rare, with a prevalence of approximately 2-3%. Beaver tail liver, which is also called sliver of liver, is a rare variant of liver morphology. Sometimes elongated left lobe of the liver can extend laterally across the midline to contact and often surround the spleen. Here, we present a case of Morgagni hernia with beaver tail liver diagnosed in a 46-year-old female.

## Case presentation

**Figure 1:**
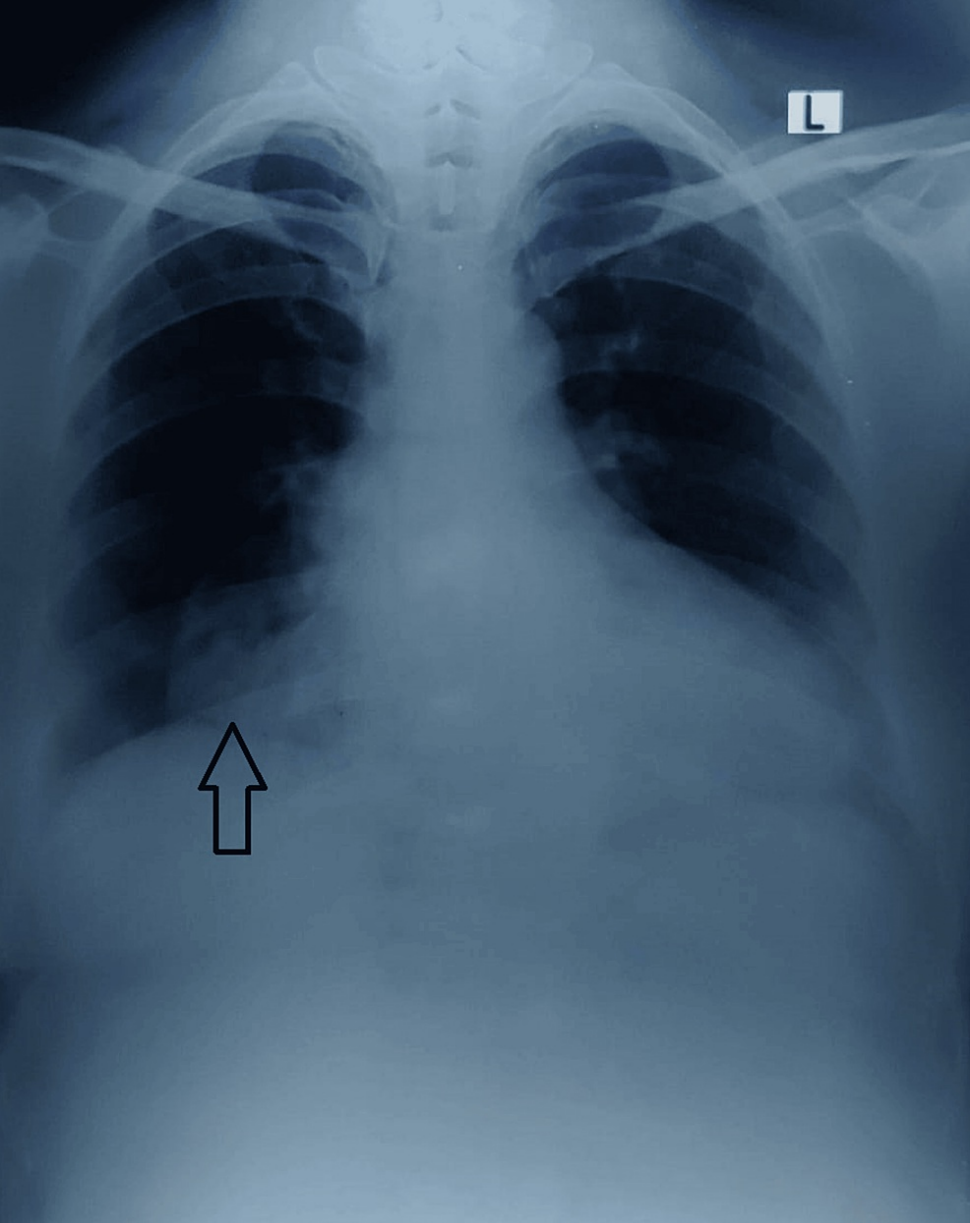
Chest X-ray posteroanterior view - right lower zone, para-cardiac well-circumscribed structure suggestive of a cyst with air-fluid level inside

A 46-year-old female from Karnataka complained of fever with chills and lower back aches for seven days. No history of cough, breathlessness, hemoptysis, chest pain, vomiting, or diarrhea was present. The patient had a previous history of similar complaints in the past with intermittent lower back aches, which have been on and off for four years. The patient had no other comorbidities. She was a non-smoker and non-alcoholic. Biomass gas exposure for 20 years was present as she cooked food with firewood. She was a housewife by occupation. She had no history of trauma or surgeries in the past. General physical examination was unremarkable with a BMI of 32. Respiratory system examination showed hyper-resonant percussion notes in the right side mammary area. Vocal resonance and vocal fremitus were reduced in the mammary area. Auscultation showed decreased breath sounds in the mammary area of the right side of the chest with normal vesicular breath sounds in all other areas. On per abdominal examination, there was a flat abdomen. Umbilicus was central in position. There was tenderness in the right hypochondriac and epigastric regions. No guarding or rigidity was noted. On examining the cardiovascular system, apical impulses could not be palpated and normal heart sounds were heard, with cardiac murmurs. Other systems examination was normal. Routine blood investigations were done, which revealed hemoglobin of 11.6%, total WBC of 6270 cells, and hematocrit of 33.1%. The renal function test was within normal limits (creatinine = 0.7 mg/dl). Chest X-ray showed a right lower zone para cardiac well-circumscribed structure suggestive of an ill-defined cavity with air-fluid level inside (Figure 1).

**Figure 2:**
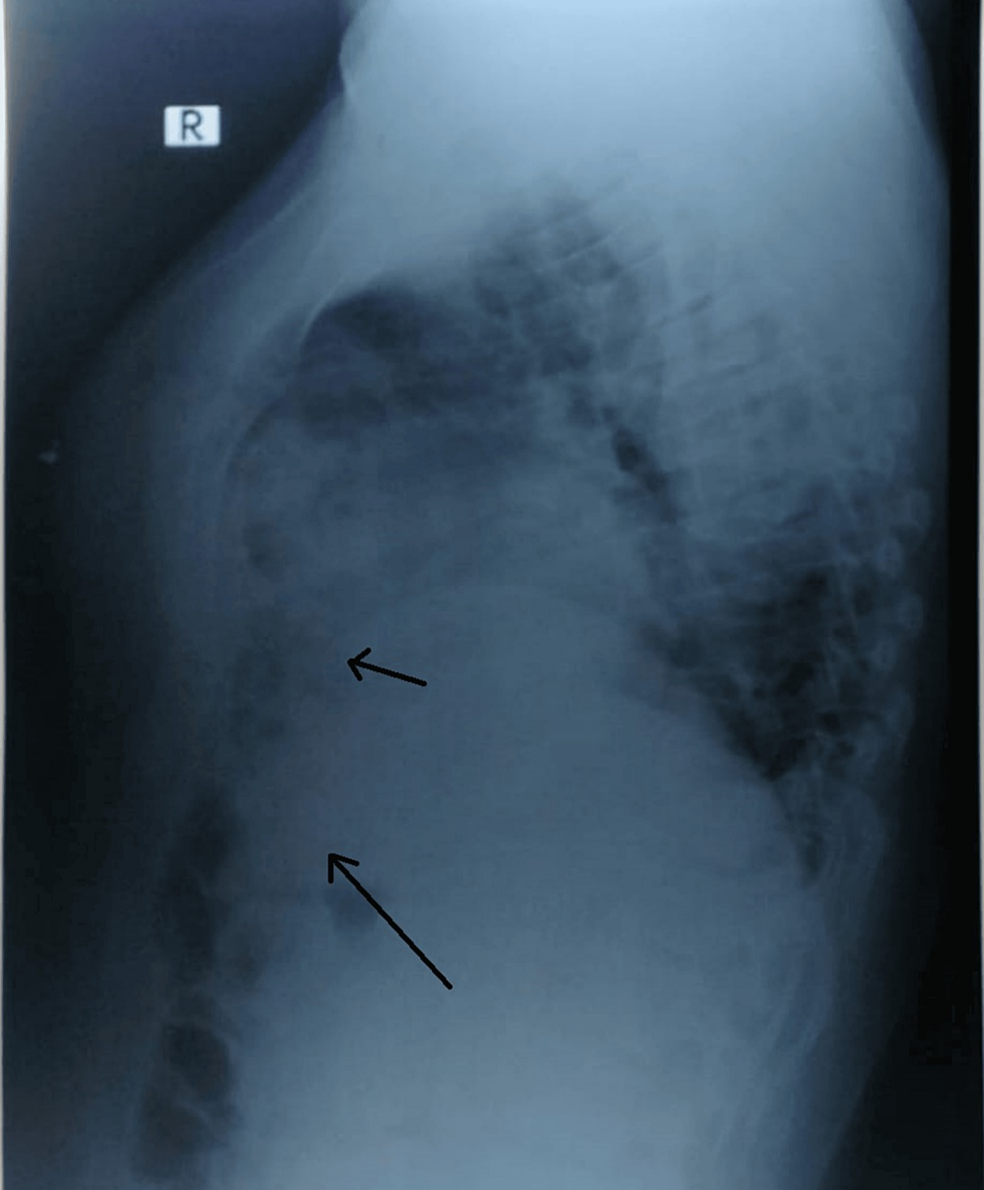
Right lateral view - herniation of intestine into the anterior mediastinum through the diaphragm

Right-lateral chest X-ray was taken to confirm the lesion and its location, suggestive of herniation of intestinal loops into the anterior mediastinum through the diaphragm (Figure 2).

CT of the chest (plain) was performed suggestive of a hernia in the anterior aspect of the diaphragm measuring 3.5 x 3.3 cm at the level of D9 vertebral body with transverse colon and omentum as its contents, ascending upwards into anterior and superior mediastinum for a length of 13 cm causing a shift of cardia posteriorly and to the left (anterior midline diaphragmatic hernia - Morgagni hernia). A hyperdense lesion (Hounsfield unit = 64) measuring 1.3 x 1.8 cm was noted in segment seven of the right lobe of the liver, suggestive of a complex cyst. Beaver tail was noted in the liver (Video 1).

**Video 1:** Morgagni hernia - transverse section lung window

The coronal section of the chest showed an anterior midline defect of the diaphragm with omentum and transverse colon herniating into the thorax (Video 2).

**Video 2:** Morgagni hernia - coronal section

A sagittal view of the CT thorax showed Morgagni hernia in the anterior mediastinal space with the cardia being pushed posteriorly (Video 3).

**Video 3:** Morgagni hernia - Sagittal view

The transverse section of the mediastinal window of the chest showed an air-filled transverse colon in the anterior mediastinum (Video 4).

**Video 4:** Morgagni hernia - transverse section mediastinal window

## Discussion

The first description of Morgagni hernia was by Comer et al. in 1769 as the rarest of congenital diaphragmatic hernias (2-3%) [2]. They usually present in younger age groups with respiratory symptoms [3]. Incidental detection of this condition in adults is very rare [4]. In this case, the patient had lower backache and no other gastrointestinal symptoms. The respiratory and cardiothoracic systems get affected because the intestinal contents herniating through the diaphragm shift the position of the cardia and the lower lobes of the lungs, which may have implications such as repeated cough and infections. Symptomatic hernias are usually detected in an early age group. Surgical reduction of the hernia with mesh repair is recommended even though the patient is asymptomatic to avoid strangulation of the herniated contents in the future, especially in this patient who also had a beaver tail liver. Since the patient is febrile and requires bed rest at the moment, the patient will be posted for surgery once the patient is fit for surgery. The plan of management for this patient would be a surgical reduction of the herniated contents back into the abdomen and insertion of mess into the hernia (hernia repair), which can be done with either laparoscopy or open surgery.

## Conclusions

Morgagni hernia is a rare type of congenital diaphragmatic hernia that is usually identified in childhood but rarely seen in adults. It can present with symptoms of gastrointestinal obstruction or acute chest symptoms or can even be asymptomatic. One thing we should keep in mind is the restriction it causes to the lungs as a result of the herniated contents occupying the thoracic cavity and reducing the lung capacity. Diagnosis is done by contrast CT of the chest and treatment is primarily surgical repair of the hernia. This can be done either transthoracically or transabdominally. It is usually advised that surgical repair should be done even in asymptomatic cases, as in this case, to avoid obstruction of the intestine or worsening of the hernia that is pulling the abdominal contents into the thorax. This research addresses the importance of detailed systemic examination in patients and having a high index of suspicion would help in the early diagnosis of rare medical conditions. It adds to the existing literature as this case had a varying clinical presentation.

## References

[1] Mohamed M, Al-Hillan A, Shah J, Zurkovsky E, Asif A, Hossain M (2020). Symptomatic congenital Morgagni hernia presenting as a chest pain: a case report. J Med Case Rep.

[2] Comer TP, Clagett OT (1966). Surgical treatment of hernia of the foramen of Morgagni. J Thorac Cardiovasc Surg.

[3] Loong TP, Kocher HM (2005). Clinical presentation and operative repair of hernia of Morgagni. Postgrad Med J.

[4] Arora S, Haji A, Ng P (2008). Adult Morgagni hernia: the need for clinical awareness, early diagnosis and prompt surgical intervention. Ann R Coll Surg Engl.

